# Direct Conversion of Food Waste Extract into Caproate: Metagenomics Assessment of Chain Elongation Process

**DOI:** 10.3390/microorganisms9020327

**Published:** 2021-02-05

**Authors:** Simona Crognale, Camilla M. Braguglia, Agata Gallipoli, Andrea Gianico, Simona Rossetti, Daniele Montecchio

**Affiliations:** Water Research Institute, National Research Council of Italy (IRSA-CNR), Via Salaria, km 29.300, 00185 Roma, Italy; crognale@irsa.cnr.it (S.C.); gallipoli@irsa.cnr.it (A.G.); gianico@irsa.cnr.it (A.G.); rossetti@irsa.cnr.it (S.R.); montecchio@irsa.cnr.it (D.M.)

**Keywords:** medium chain fatty acids, microbial chain elongation, caproate, waste valorisation, metagenomics

## Abstract

In a circular economy strategy, waste resources can be used for the biological production of high added-value substances, such as medium chain fatty acids (MCFAs), thus minimising waste and favouring a sustainable process. This study investigates single-stage fermentation processes for the production of MCFAs in a semi-continuous reactor treating the extract of real food waste (FW), without the addition of external electron donors. Two sequential acidogenic fermentation tests were carried out at an organic loading rate (OLR) of 5 and 15 gCOD L^−1^d^−1^ with a hydraulic retention time of 4 days and pH controlled at 6 ± 0.2. The highest level of caproate (4.8 g L^−1^) was observed at OLR of 15 gCOD L^−1^d^−1^ with a microbiome mainly composed by lactate-producing *Actinomyces*, *Atopobium*, and *Olsenella* species and caproate-producing *Pseudoramibacter*. Metagenomic analysis revealed the presence of key enzymes for the production of lactate, such as lactate dehydrogenase and pyruvate ferredoxin oxidoreductase, as well as several enzymes involved in the reverse β-oxidation pathway, thus suggesting the occurrence of a lactate-based chain elongation process.

## 1. Introduction

Recent applications of anaerobic digestion technology are moving towards the generation of high added-value products that includes carboxylic acids such as succinic, lactic, and volatile fatty acids (VFAs) [1]. The fermentation products comprise a mixture of straight-chain fatty acids, resulting from sugars and amino acids fermentation and β-oxidation of fatty acids, usually referred to as short-chain (C2–C4), medium-chain (C6–C10), long-chain (C12–C18), and a group of branched-chain fatty acids [2]. Recently, particular interest has grown in the biological production of medium chain fatty acids (MCFAs) from renewable resources as a key step to tackle societal dependence on fossil fuels [3,4,5]. These chemicals can be used as precursors of liquid biofuels or commercial chemicals [6]. Caproic acid, for example, has a wide range of applications in animal feed additives, green antimicrobials, plant growth promoters, and fragrances [7,8,9,10]. Usually, caproic acid is obtained from coconut or palm [11] and has a high economic market value [12,13]. It derives from chain elongation (CE) reactions in which short chain fatty acids (SCFAs) are converted to MCFAs by using mainly ethanol or lactate as an electron donor [14]. One of the strategies for enhancing MCFAs production is to promote the acidogenic process (i.e., fermentation of soluble carbohydrates and aminoacids) by inhibiting the methanogenesis through optimised reactor operation conditions and feeding patterns [15]. The use of organic waste implies the presence of soluble substrate, in particular sugars, that can favour the primary fermentation generating ethanol or lactate (mainly from kitchen wastes [16]) avoiding the addition of an external electron donor for the CE process. The CE process is mediated by microorganisms through the reverse β-oxidation (RBO) pathway [17]. *Clostridium kluyveri* was the first described microorganism able to elongate short chain carboxylates (e.g., acetic acid) to caproic acid via RBO by using ethanol as an electron donor [18]. Recently, evidences of lactate-based chain elongation [19] via few microorganisms (e.g., *Megasphaera elsdenii*, *Megasphaera hexanoica*, *Pseudoramibacter alactoliticus*, and *Ruminococcaceae* bacterium CPB6) involved in this process has been reported [4,12,20]. There have been a number of reports on the capability of microorganisms to use sugars, methanol, amino acids, or hydrogen as energy source for CE [21,22,23]. In terms of microbial metabolic pathways, the CE reactions with ethanol or lactate follow a similar RBO path as described in *C. kluyveri* [14,24]. As recently highlighted, several enzymes are involved in this metabolic pathway in mixed microbial cultures [25,26]. In particular, alcohol dehydrogenase or lactate dehydrogenase are required for the initial conversion of ethanol or lactate into acetyl-CoA, respectively. The acetyl-CoA enters cyclic RBO and is transformed to butyryl co-A due to the sequential activity of acetyltransferase, 3-hydroxyacyl-CoA dehydrogenase, enoyl-CoA hydratase, and butyryl-CoA dehydrogenase enzymes. Lastly, butyryl-CoA:acetate CoA-transferase mediates the formation of butyryl-CoA to butyrate and caproate following a second RBO cycle [27].

In the last decade, several studies focused on the production of MCFAs from waste biomass allowing their valorisation and satisfying environmental and economic demands. Some waste biomass, such as yeast-fermentation beer, lignocellulosic stillage, and wine lees, were tested as feedstock for the production of MCFAs as they usually already contain ethanol or lactate and SCFAs [4,28,29]. In some cases, yeast strains (e.g., *Kluyveromyces* sp.) were engineered for improving sugar utilisation and ethanol fermentation [30]. In other studies, a feedstock requiring the addition of external electron donor (e.g., organic fraction of municipal solid waste, switchgrass) was used in a two-stage bioreactor allowing the separation of hydrolysis/acidification step and CE [31,32,33]. Recently, in order to reduce costs associated with the addition of ethanol/lactate or SCFAs, the use of acid whey and food waste (FW) were investigated in both one- and two-stage processes, since these feedstocks are capable of producing both electron donors (EDs) and acceptors (EAs) for MCFAs production [34,35,36,37,38]. The separation and recovery of caproate remain still challenging due to the complex nature of the fermentation broth. The most promising methods, investigated at a lab-scale, are solvent extraction, adsorption, and membrane processes, but a mature technology is still missing.

The biological production of caproate (up to 10 g L^−1^) from FW was obtained during the operation of a single-stage anaerobic reactor and was found to be most likely driven by *Ruminococcaceae* bacterium CPB6 and *Clostridium* sp.MT1 [35]. Reddy et al. [15], showed a high caproate production (8.1 g L^−1^) from FW by using a pure culture of *C. kluyveri* together with a chain-elongating enriched culture. Before these, few studies investigated the production of MCFAs from FW without analysing the microbial component responsible for the process [17]. Recently, Contrera-Davilas et al. [39] investigated FW fermentation highlighting the capability to produce up to 4.5 g L^−1^ of lactate and 5.4 g L^−1^ of caproate by *Lactobacillus* spp. and *Caproiciproducens* spp., respectively. In this paper, as in [39], we denote n-caproate as undissociated n-caproic acid and dissociated n-caproate together.

The composition and amount of the fermentation products and microorganisms involved in the fermentation process represent the main issue receiving increasing attention in recent years. Correlation between the microbial community structure and the fermentation process performances is still a field full of gaps, especially in the case of semi-continuous long term processing with real waste stream. Thus far, few studies have focused the attention on the use of real FW.

This study aims at investigating the long-term performances and viability of the fermentation/fatty acids CE processes in a semi-continuous reactor fed with the liquid extract of real FW. The qualitative, predictive relationships between the complex microbial community structure and the fermentation outputs were evaluated. Moreover, successional changes in prokaryotic and eukaryotic communities and a metagenomics focus on the chain-elongating microbiome provided.

## 2. Materials and Methods

### 2.1. Substrate

Food waste (FW) was collected from the cafeteria of the research area “Roma 1” of the National Research Council. The cafeteria serves approximately 300 researchers per day and produces approximately 400 kg of FW per week, which consisted of mixed raw and cooked food such as cheese (15%), bread and pasta (15%), and fruit and vegetable peelings (70%). FW was collected in multiple acquisitions and was manually screened in order to maintain such fixed composition typical of household FW. Successively, sorted scraps were firstly manually chopped and then shredded (particle size below 1 cm) by a lab-scale knife mill, prior to being stored at −20 °C.

The liquid/solid separation phase on FW (diluted with tap water in a weight ratio 1:4) was performed with a bench scale centrifuge Rotanta 460 (Hettich, Germany) operating at 4600 rpm for 10 min. The liquid phase (“extract”) was then filtered through a 0.5 mm mesh sieve to remove the coarse residue particles.

### 2.2. Analytical Methods

Total and volatile solids were determined according to standard methods (APHA, 1998). The pH was detected by in situ pH probe INPRO4800I/SG/120 (Mettler Toledo, Milan, Italy). Soluble (CODsol) and total COD, measured in duplicates, were determined by means of the COD Cell Test (Spectroquant Merck, Darmstadt, Germany)(EPA method 410.4). Soluble proteins and carbohydrates were measured on filtered liquid samples (glass filters GF/C Whatman, 1.2 μm porosity), protein content was determined by means of a modified Lowry method [40], while carbohydrates determination was based on a modified DuBois method [41,42]. Total proteins content was estimated by multiplying the total organic nitrogen content by a factor of 6.25.

The biogas composition was measured using a PerkinElmer Auto System Gas Chromatograph equipped with a thermal conductivity detector (TCD). Volatile fatty acids (VFAs) were analysed by injecting 1 μL of filtered (0.22 μm porosity) liquid sample into a Perkin Elmer Auto System gas-chromatograph equipped with a FID detector (flame ionisation detector).

### 2.3. Acidogenic Step with Liquid Extract

For each fermentation trial, a continuous reactor with a working volume of 3 L was operated anaerobically at a mesophilic temperature of 37 ± 2 °C. Two sequential acidogenic fermentation tests were carried out at an organic loading rate (OLR) of 5 and 15 gCOD L^−1^d^−1^, respectively, and HRT of 4 days. The pH in the reactor was controlled every day and adjusted to 6 ± 0.2 by adding a solution 2.7 M of Na_2_CO_3_.

For the start-up, the reactor was initially filled with anaerobic inoculum deriving from a digester of a local WWTP and acclimated one week progressively to FW extract, used then as the substrate for fermentation.

Hydrogen was measured online at T = 0 °C and p = 1 atm (µFlow, Bioprocess Control, Lund, Sweden), after the CO_2_-fixing unit (CO_2_ trap, filled with NaOH 3 M solution).

### 2.4. Calculations

Process performances (in terms of acidification degree, production rate, and specificity) was assessed after reaching quasi-steady state or steady state conditions in terms of stable fatty acids production.

In the fermentation reactor, acidification degree (%) was calculated as the ratio between VFAs (from acetate to caproate) concentration (in terms of COD, mg L^−1^) and soluble COD concentration (mg L^−1^):

(1) $$acidification\;degree\;\left(\%\right)=(COD_{vfa}/COD_{sol})\times 100$$

(2) $$Caproate\;production\;rate\;\left(mmolC\;L-1d-1\right)=\frac{\left(Cp\;\times \;6\right)\times Q}{V}$$

where:

*Cp* = concentration of caproate in the reactor, mM;
6: number of carbons in caproate;
*Q* = effluent flow rate, L d^−1^;
*V* = volume of the reactor, L;
  (3) $$specificity\;\left(\%\right)=\;\frac{c_{p}}{\sum_{i=1}^{n}c_{i}}\;\times 100$$
*c_p_* = product concentration in the reactor, gCOD L^−1^;
*c_i_* = concentration of all detected VFAs (without ethanol and lactic acid), gCOD L^−1^.

### 2.5. Catalysed Reporter Deposition-Fluorescence In Situ Hybridisation (CARD-FISH)

Anaerobic sludge samples (4.5 mL) were taken over the reactor operation at 12 different sampling times and immediately fixed in formaldehyde and ethanol (2% and 50% *vol/vol* final concentration respectively) and stored at −20 °C. Small aliquots of biomass were disaggregated by vortexing the sludge samples in the presence of glass beads for a few minutes and then used for the CARD-FISH analysis followed the procedure described in Matturro et al. [43]. The analysis was performed using the oligonucleotide probes EUB338mix (equimolar concentrations of EUB338, EUB338-II, and EUB338-III) for total Bacteria, LGC354abc for *Firmicutes*, and HGC69a for *Actinobacteria*. Probe details and conditions are reported in probeBase (http://www.microbial-ecology.net/probebase/) (accessed on 5 February 2021). After hybridisation, total cells were stained with Vectashield Mounting Medium^®^ with DAPI (Vector Labs, Segrate, Italy).

### 2.6. DNA Extraction

A small aliquot of anaerobic sludge samples (2 mL) was used for DNA extraction. A centrifugation at 15,000 rpm for 2 min was required in order to obtain pellet for subsequent DNA extraction with a DNeasy PowerSoil Pro Kit (QIAGEN, Antwerp, Belgium). A Nanodrop 3300 (Thermo Scientific, Monza, Italy) was used in order to assess DNA quality (1.6 < A260/280 < 1.8 and A260/230 > 2).

### 2.7. High-Throughput rRNA Genes Sequencing and Bioinformatic Analysis

The extracted DNA was utilised as a template for the amplification of the V1-V3 region of 16S rRNA gene of bacteria (27F 5′-AGAGTTTGATCCTGGCTCAG-3′; 534R 5′-ATTACCGCGGCTGCTGG-3′) and the V4 region of 18S rRNA gene of eukaryotes (Eu565F: 5′-CCAGCASCYGCGGTAATTCC-3′; Eu981R: 5′-ACTTTCGTTCTTGATYRA-3′) following the procedure described in Crognale et al. [44] and Ul-Hasan et al. [45]. All PCR reactions were carried out with a Phusion High-Fidelity PCR Master Mix (Thermo Fisher Scientific, Waltham, MA, USA). The purification of sequencing libraries was performed according to the bead protocol of the Agencourt^®^ AMpure XP (Beckmann Coulter, Milan, Italy). The Qubit 3.0 Fluorometer (Thermo Fisher Scientific, Waltham, MA, USA) was used for quantifying library concentration. With the MiSeq Reagent kit v3, 600 cycles (Illumina, San Diego, CA, USA) was used for paired end sequencing (2 × 301bp) on a MiSeq platform (Illumina, San Diego, CA, USA). The Phix control library was spiked at a concentration of 15%.

The raw sequences was firstly quality checked with fastqc and then analysed using QIIME2 v. 2018.2 [46]. The QIIME2 plugins demux (https://github.com/qiime2/q2-demux 10/02/2018) and cutadapt (https://github.com/qiime2/q2-cutadapt 02/12/2017) were used for demultiplexing reads and removing primer sequences. The demultiplexed reads were processed with the DADA2 pipeline in order to identify amplicon sequence variants (ASVs) [47,48]. The reads were subsampled and rarefied at the same number for each sample by using the feature-table rarefy plugin [49]. The taxonomy was assigned to ASVs using a pre-trained naïve-bayes classifier based on the 16S rRNA or 18S rRNA gene database at a 99% similarity of the SILVA132 release [50]. High-throughput sequencing of the V1-V3 region of the bacterial 16S rRNA gene yielded a total of 116,700 sequence reads after quality control and bioinformatic processing that resolved into 535 ASVs. The taxonomic assignment of 21 major ASVs was additionally carried out by the BLASTn algorithm [51]. The 16S and 18S rRNA gene sequences were deposited in the GenBank database under the accession numbers MW420990-MW421300 and MW433275-MW433567, respectively.

### 2.8. Metagenome Sequencing and a Genome-Centric Analysis

A small aliquot (30 µL) of DNA from a sample taken at the end of operation at an OLR of 15 gCOD L^−1^d^−1^ was sent to DNASense laboratories (Aalborg, Denmark) for metagenomics analysis. DNA concentration and quality were evaluated using Qubit dsDNA HS kit and TapeStation with the Genomic ScreenTape (Agilent Technologies, Milan, Italy), respectively. The sequencing library was prepared using the NEB Next Ultra II DNA library prep kit for Illumina (New England Biolabs, Beverly, MA, USA) following the manufacturer’s protocol. Library concentration was measured in triplicate using the Qubit dsDNA HS kit and library size estimated using TapeStation with D1000 HS ScreenTape. The sample was paired end sequenced (2 × 301bp) on a MiSeq (Illumina, San Diego, CA, USA) using a MiSeq Reagent kit v3 with 600 cycles (Illumina, San Diego, CA, USA), following the standard guidelines for preparing and loading samples on the MiSeq. Raw Illumina reads were filtered for PhiX using Usearch11 [52] subsequently trimmed using Cutadapt v. 2.10 [53]. Forward and reverse reads were used to perform de novo assembly in megahit v. 1.2.9. Bins were subsequently extracted in mmgenome2 v. 2.1.3 and bins were quality-assessed with CheckM v. 1.1.3 [54]. A classification of bacterial bins was performed with the Genome Taxonomy Database toolkit (GTDB-TK) v. 1.3.0 [55]. Genome annotations of bacterial and archaeal genomes were conducted with Prokka v. 1.14.6 [56]. The joint reads were also annotated according to the COG database to perform a more detailed analysis of the functional genes [57]. Average nucleotide identities (ANI) were calculated using FastANI v. 1.32 [58,59]. This Whole Genome Shotgun project has been deposited at DDBJ/ENA/GenBank under the accession JADOBB000000000, JAEAMH000000000-JAEAMQ000000000 (bioproject PRJNA675427).

### 2.9. Statistical Analysis

The chemical data were incorporated into a Non-metric MultiDimensional Scaling ordination plot (NMDS) in order to graphically synthesise the Euclidean dissimilarity between samples by using PAST software (PALAEONTOLOGICAL STATISTICS, ver. 2.17) [60]. The NMDS ordination of chemical and microbiological data was performed by using a vector-fitting procedure, showing a direct proportion of arrow’s length with the correlation between NMDS-axes and the analysed variables. This approach highlighted the variation pattern of each variable distinguishing the samples [61,62]. Chemical data and relative abundances of the microbial taxa revealed by 16S rRNA gene high-throughput sequencing (only genera ≥5% of total reads were considered) were normalised by log(X + 1).

## 3. Results

### 3.1. Primary Fermentation and Chain Elongation of FW Extract

The liquid extracts obtained from the solid-liquid separation unit have been characterised (Table 1) in terms of COD, solids, and soluble organic matter and appropriately diluted in order to achieve the desired OLR. The total COD (totally constituted by soluble substance) was 21 and 60 g L^−1^ for the OLR 5 and OLR 15 test, respectively. The feed soluble organic matter was mainly constituted by free carbohydrates (65–70%). Biogas was mainly comprised of hydrogen and carbon dioxide, since methane production was always negligible.

**Figure 1:**
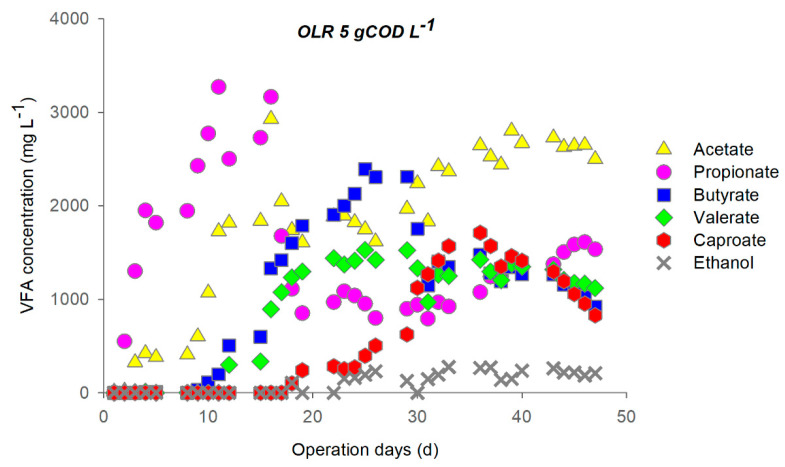
Profile of metabolites during operation at OLR 5 (feeding cycle: 4 days, stop feeding cycle: 3 days).

During primary fermentation at OLR 5, the soluble carbohydrates load (3.3 ± 0.2 gCOD L^−1^d^−1^) was completely removed, while the average removal of proteins reached 80 ± 5% after the third cycle (Figure S1). Over the first two feeding cycles, the fermentation was propionic-like [63], most likely due to the inoculum, with an observed maximum propionate concentration corresponding to 3300 mg L^−1^ (Figure 1). The system shifted then to butyric-like fermentation and CE started to take place at the end of the third cycle.

Low ethanol concentration was detected starting from the fourth cycle (always below 300 mg L^−1^), when caproate was also produced. The latter sharply increased at the end of the fifth cycle, when the system appeared to reach semi steady-state conditions. Maximum caproate concentration (~1700 mg L^−1^) was achieved between the end of the fifth cycle and the beginning of the sixth one. The simultaneous butyrate consumption and caproate production was observed at the beginning of the fifth cycle.

It is important to note that, during the short (3 days) cycle of stop feeding, no changes in metabolite concentration and in soluble COD concentration were observed. This outcome suggests that under these operational conditions, since the readily biodegradable substrate was consumed and the fermentation process was completed, the CE pathways interrupted, too. This was supported by the H_2_ yield drop (~90%) observed over the stop feeding days.

During the sixth and seventh cycle, butyrate and caproate decreased while propionate concentration increased suggesting a shift of the fermentation process to the propionic-like one, thus reducing the EAs available for caproate formation.

With regard to the process performance, during the last cycles of the operation the acidification degree was 85 ± 7%, while the conversion efficiency of soluble COD into caproate reached a maximum value of 16.5% (after 30–35 operation days).

**Figure 2:**
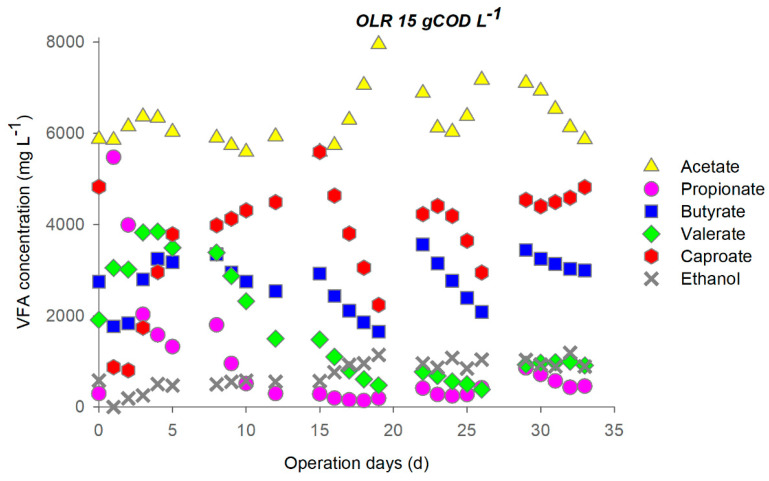
Profile of metabolites during operation at OLR 15 (feeding cycle: 4 days, stop feeding cycle: 3 days).

The reactor operated at OLR 15 gCOD L^−1^d^−1^, the daily sugars load was 9 ± 1 gCOD L^−1^d^−1^, and it was almost completely removed. Protein removal remained stable at 85% during the first 4 cycles but decreased at the end of the test (Figure S2). During the first days of operation, chain elongation occurred contemporarily with the decrease of the propionate (Figure 2). Indeed, during the first week, fermentation quickly switched from propionic-like to butyric-like, with a decrease of propionate to 6.3 gCOD L^−1^ and a concurrent rise of butyrate to 2.4 gCOD L^−1^ (14 mmol L^−1^). Caproate increased by 25 mmol L^−1^, suggesting that an additional butyrate production of 39 mmol L^−1^ took place during the first cycle (out of which, 25 mmol L^−1^ were upgraded to caproate). In the second cycle, caproate increased to 4800 mg L^−1^, with a concurrent butyrate reduction, indicating an efficient use of the EDs.

It is worth observing that, during the 3 days of stop feeding, the caproate was continuously produced whereas the metabolites other to butyrate remained nearly constant (Figure 2).

The CE process appeared to reach a stable configuration only over the fifth cycle, when caproate and most of the metabolites reached a stable concentration. On the other hand, both caproate and butyrate decreased during the third and fourth cycle. Concerning process performances, it is worth noting that during the stable phase around 18% of the influent fermentable COD was converted into caproate.

Changes in pH (Figure S3) and the metabolites profile (Figure 1 and Figure 2) indicated that during the feeding cycle, a rapid acidification of the medium occurred due to fermentation from sugars that released protons. The addition of Na_2_CO_3_ was hence necessary to maintain the pH around 6 ± 0.2 during the day. The synthesis of caproate during the 3 days of stop feeding cycle, observed at a high OLR, seemed to be linked to lactate as the electron donor because of the evident pH increase due to the proton consuming behaviour of lactate-based chain elongation [39].

### 3.2. Bacterial Community Composition

CARD-FISH analysis revealed *Firmicutes* and *Actinobacteria* as the main bacterial components in both reactors with a marked dominance of cells affiliated with *Actinobacteria* (~90% of total DAPI stained cells) in samples taken from the system operating at a high ORL 15 gCOD L^−1^d^−1^ (Figure S4).

**Figure 3:**
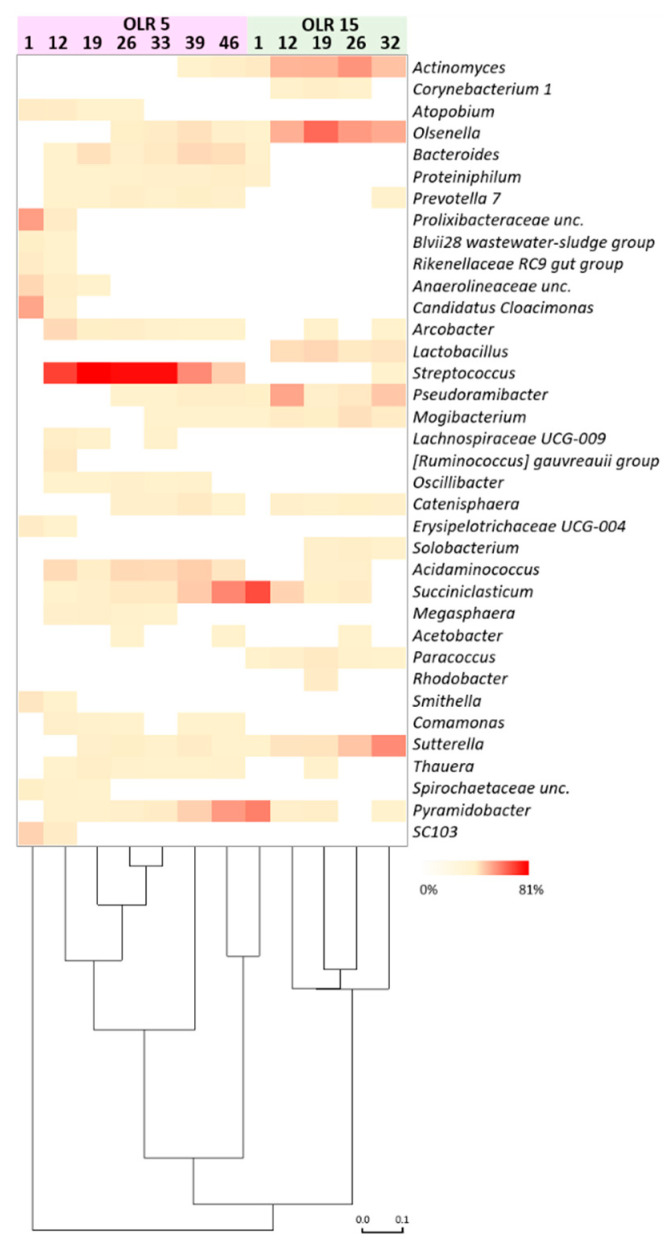
Relative abundance of microbial genera (≥1% in at least one sample) during the reactors operation. Bray Curtis statistics was used for clustering the samples according to the process parameters.

Consistent with CARD-FISH analysis, high-throughput sequencing showed in both reactors the predominance of reads mostly affiliated with phyla *Actinobacteria* and *Firmicutes*, followed to a minor extent by *Bacteroidetes*, *Proteobacteria*, and *Synergistetes*. The biomass in the reactor operating at OLR of 5 gCOD L^−1^d^−1^ was dominated by *Streptococcus* species accounting for 12.1–81% of total reads (Figure 3).

Interestingly, an increase of reads affiliated with *Succiniclasticum* (0–36.6%) and *Pyramidobacter* (0–30.4%) was observed during the concomitant decreasing trend of the relative abundance of reads belonging to *Streptococcus* (from 32 to 45 day of operation). Sequences belonging to *Actinomyces*, *Olsenella*, *Psudoramibacter*, *Acidaminococcus*, and *Sutterella* were found to a minor extent. The sequencing results of the biomass in the reactor that operated at 15 gCOD L^−1^d^−1^ showed a large occurrence of reads mainly affiliated with the genera *Actinomyces* (range 3–31.9% of total reads), *Olsenella* (0.3–46.1%), *Lactobacillus* (0–9.4%), *Pseudoramibacter* (1–26.5%), *Mogibacterium* (0.1–6.5%), and *Sutterella* (0.2–35%) (Figure 3). A marked decrease of reads belonging to the genera *Succiniclasticum* (55.5–0%) and *Pyramidobacter* (38.6–0.1%) was observed over the operation.

**Figure 4:**
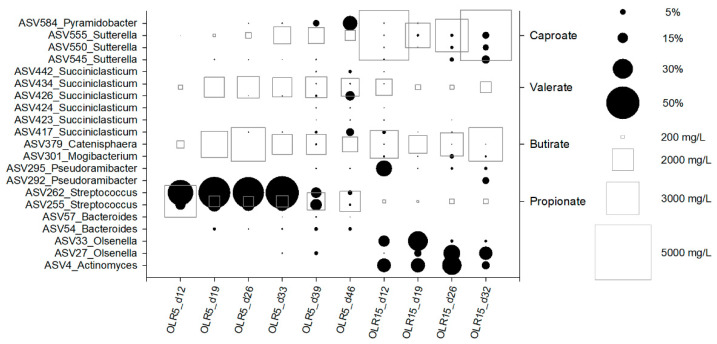
Bubble plot depicting the relative abundance (as % of total reads) of the main ASVs (black circles) and the concentration of propionate, butyrate, valerate and caproate (mg L^−1^) (white squares) during reactors operation.

The microbial succession during reactors operation was also apparent at the level of amplicon sequence variants (ASVs) (Figure 4, Table 2). A total of 21 ASVs counted between 55% and 88% of total reads obtained from samples. In the reactor operating at OLR of 5 gCOD L^−1^d^−1^, ASV255 and ASV262 prevailed over the operation and were replaced by several ASVs ascribable to genera *Succiniclasticum* and *Pyramidobacter* at the end of the operation (day 39, Figure 3 and Figure 4). In contrast, *Actinomyces*, *Olsenella*, *PseudoramibacterI*, and *Sutterella* ASVs prevailed in the reactor operated at 15 gCOD L^−1^d^−1^ concomitantly with the highest production of caproate.

### 3.3. Eukaryotic Community Composition

The high-throughput sequencing of the 18S rRNA gene was aimed at ascertaining a possible role of eukaryotes in the VFA production process. A total of 1,042,476 sequence reads of the V4 region of the eukaryotic 18S rRNA gene was yielded and resolved into 385 ASVs. The reactor operating at low OLR was inhabited mainly by *Cercozoa* and *Eukaryota* not identified at the lowest taxonomic levels (Table S1). A relative low abundance of Fungi, affiliated with *Ascomycota* and *Basidiomycota* was observed. In contrast, *Ascomycota* affiliated with *Geotrichum*, *Yarrowia*, *Hanseniaspora*, and *Trichosporonaceae* belonging to *Basidiomycota* were the main eukaryotic components in the reactor at a high OLR. Remarkably, on average 0.1% of total reads were affiliated with genera *Kluyveromyces*, *Pichia*, and *Saccharomyces*.

### 3.4. Genome Bin Statistics and Metagenome

Metagenomic sequencing generated a total of 9,126,878 raw reads that were base quality and phiX-filtered, obtaining 9,041,616 trimmed reads before the de novo metagenome assembly. The number of contigs and total length of the 11 individual genomes (genome bins) extracted from sample metagenome are shown in Table 3. The shortest contig length necessary to cover 50% of the genome was likewise determined along with an evaluation of the content of GC-nucleotides. Furthermore, the degree of genome completeness (%) and contamination (%) of each bin was estimated using unique marker genes. The metagenome assembled well with relatively large contigs for the most abundant genome bins. A total of 11 genome bins were extracted based on the differential genome abundance and using a kmer-based tSNE approach (t-distributed stochastic neighbor embedding).

Overall, all genome bins are highly complete (88–100%) and contain very low levels of contamination (0–1.9%) as ascertained by the presence of CheckM marker genes. All genome bins have been classified using the Genome Taxonomy Database (GTDB) (Table 3), with the three most abundant species being *Actinomyces succiniciruminis*, *Pseudoramibacter alactolyticus*, and *Olsenella umbonata*. The number of predicted coding sequences (CDS) ranged from 1699 to 2665. For each bin, a high portion of CDS were classified in COG functional categories (from 30.2% to 42.7%) and around 900 CDS were annotated as hypothetical protein.

**Figure 5:**
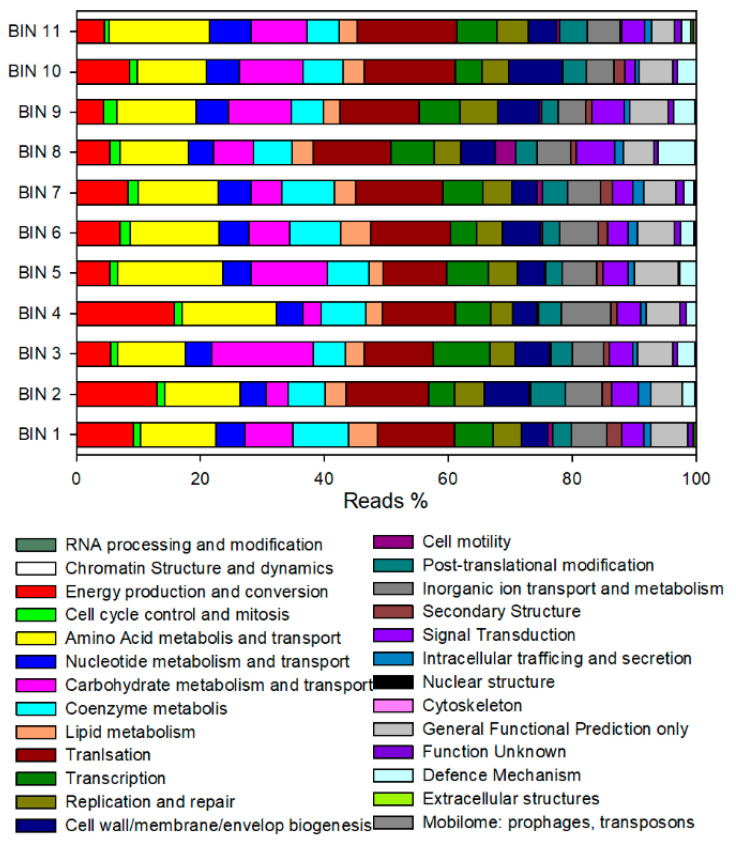
Distribution of predicted reads in the COG classification.

The major functional categories of the extracted bins were similar (Figure 5). The energy production and conversion, amino acid transport and metabolism, carbohydrate metabolism and transport, lipid metabolism, and translation were the most abundant functions identified. The relative abundance of reads assigned to cell motility, nuclear structure, cytoskeleton, extracellular structures, and mobilome were the lowest among all the bins.

A manually curated analysis of CDS annotated in “energy production and conversion” and “lipid metabolism” categories was performed for exploring the presence of genes involved in bioprocessing of lactate oxidation to acetyl-CoA, reverse β-oxidation, hydrogen formation and energy conservation. According to Liu et al. [27], the presence or absence of key functional enzymes is schematically reported in Table 4. The bin 1, classified as *Pseudoramibacter alactolyticus*, harboured several enzymes involved in the acetyl-CoA formation (lactate racemase, lactate dehydrogenase, pyruvate ferredoxin oxidoreductase) and RBO (acetyl-CoA acetyltransferase, 3-Hydroxyacyl-CoA dehydrogenase, enoyl-CoA hydratase, butyryl-CoA dehydrogenase, butyryl-CoA:acetate CoA-transferase). Almost all bins showed lactate dehydrogenase (LDH) and pyruvate ferredoxin oxidoreductase (PFOR) enzymes. Bin 5 (*Olsenella umbonata*), bin 7 (*Mogibacterium timonense*), and bin 10 (*Prevotella*) also harboured a butyrate kinase enzyme involved in butyrate production. In addition, bin 2 (*Mesosutterella multiformis*), bin 6 (*Succiniclasticum* sp900316935), bin 8 (*Lachnospira rogosae*), and bin 11 (*Bifidobacterium thermophilum*) included enzymes involved in a succinate formation pathway (e.g., succinyl-CoA:coenzyme A transferase, succinate--CoA ligase, succinate dehydrogenase).

## 4. Discussion

Among the organic waste, FW can be widely reused for the production of high-added value products, such as MCFAS through complex microbial bioprocesses [36]. However, when working with real feedstock, several competing and intertwining biochemical pathways occur simultaneously and the produced intermediates can influence both microbial composition and final product yields. At fixed operational process parameters (i.e., pH control), the feedstock composition plays a crucial role in shaping the microbiome and process performance. The FW extract used in this study, rich in bioavailable sugars, promoted the primary fermentation by producing in situ electron donors together with VFAs useful for the chain elongation. The in situ production of lactate and ethanol is principally ascribed to the origin of the feedstock, in particular, lactic acid was found to be the main fermentation product for kitchen wastes [16,64], especially when high concentrations of easily degradable substrate are available [65]. Following ethanol, lactate is the second most investigated electron donor in CE, where part of the lactate is converted to propionate via the acrylate pathway and another part is released as CO_2_ to oxidise lactate into acetyl-CoA [29].

The conversion efficiencies of the soluble COD into caproate observed in this study, namely 16.5% and 18%, were in the range of those obtained in previous studies (13–59%) [17,66]. The maximum caproate production rate (59 mmolC L^−1^d^−1^) and concentration (9.7 mM) were slightly lower to those obtained by Contrera-Davila et al. [39] who investigated a lactate-based CE in a stirred tank reactor at uncontrolled pH, with the initial addition of mineral medium and vitamins and a chain-elongating microbial inoculum. The maximum production rate of caproate reported by Zhu et al. [19] was more than double the rate reported in the present study, however they used a fermentation pit as a unique artificial microbiome for caproate production. In contrast, the maximum production rate of caproate obtained was higher than that previously reported by Domingos et al. [66] (35 mmolC L^−1^d^−1^) with a bioreactor system treating cheese whey. Nevertheless, with regard to the carboxylic acid product specificity (product-to-VFAs ratio in terms of COD), when the best performance at OLR 15 was achieved, caproate selectivity accounted for 40 ± 1% in line with the values reported elsewhere without external ED supplementation [35,39].

In these fermenters, sugars and aminoacids fermentation brought about the yield of both EDs, ethanol and lactate, and EAs, namely acetate, butyrate, propionate, and valerate, without the need of external EDs supply. In this context, the bottleneck for the MCFAs yield is the production rate of the EDs along with the efficiency in their utilisation in the CE process. In particular, butyrate upgrading is the most efficient CE step with regard to EDs consumption [17]. In fact, butyrate elongation only requires 1/1.2 mol of ED per mole of caproate produced (with lactate/ethanol as ED), whereas acetate and EDs upgrading requires 2/2.4 and 3/3.2 moles of ED (with lactate/ethanol as ED), respectively. In this view, the experiments showed variable results however, the OLR5 test appeared to be the most effective between the fourth and fifth cycle. Indeed, the simultaneous butyrate consumption and caproate production was observed at the beginning of the fifth cycle, when butyrate decreased down to around 1 g L^−1^ (11–12 mmol L^−1^), whereas caproate increased up to around 1.2 g L^−1^ (10–11 mmol L^−1^), suggesting that caproate was entirely produced through butyrate upgrading. The OLR15 test was far less efficient in this regard in fact, during the 3 days of stop feeding, caproate was continuously produced whereas butyrate increased by 4 to 20 mmol L^−1^, suggesting that CE mostly proceeded through EDs and/or acetate upgrading. In particular, the acidification degree increased by 6–9 points, suggesting that an undetected ED (most likely lactate) had been upgraded to VFAs and finally to caproate. This is also confirmed by the constant level of ethanol observed. Furthermore, the CE process appeared to be interrupted in some phases (in particular 3 and 4) of the OLR15 test. Indeed, during the third cycle, caproate and butyrate showed a sharp decline, juxtaposed to acetate which significantly increased. It is possible to speculate that (at least) a portion of acetate was produced by ED oxidation, and that the CE process was not extended to butyrate and caproate production, thus lowering the ED consumption. Furthermore, considering that the acidification degree decreased by ~15 points in just 5 days (data not shown), with a consequential rise of the undetected soluble COD, it is very plausible that some other metabolite, most likely lactate, has been formed during the fermentation process. Similar speculations can also be done for the fourth cycle. Moreover, the propionate profile (in particular during the OLR 5 test), increasing by decreasing caproate and vice versa, indicated by the co-existence of the two competing pathways of lactate, namely (1) the RBO pathway to n-caproate and (2) the acrylate pathway to propionate.

The significant production of valerate (up to 1 g L^−1^ in the OLR 15 test) could be ascribed to amino acids fermentation [67] or propionate upgrading [17]. Nevertheless, considering that in the fifth cycle of the OLR 15 test, protein degradation even decreased (Figure S2), and it is quite likely that a good portion of valerate was produced through the latter pathway. Indeed, the presence of acetyl-CoA produced along EDs oxidation can favour the propionate elongation to valerate.

**Figure 6:**
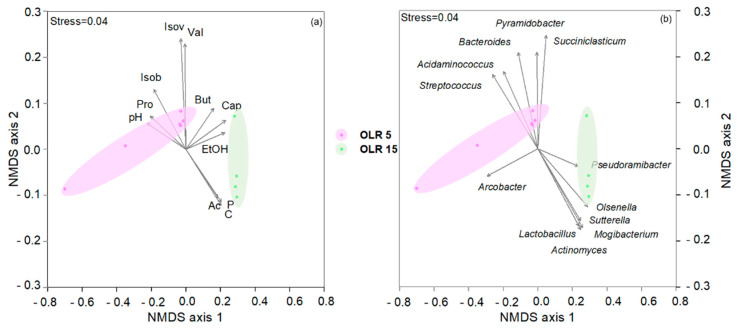
Non-metric MultiDimensional Scaling ordination plot (NMDS) ordination plots, based on Euclidean distance matrixes of log-transformed data. The vector length is proportional to the correlation between the NMDS axes and each process parameter and microbiological values. The stress value (i.e., <0.2) suggests for an accurate representation of the dissimilarity among samples. (**a**) The variation pattern of volatile fatty acids (mg L^−1^) (Ac, acetate; But, butyrate; Cap, Caproate; Isob, isobutyrate; Isov, isovalerate; Prop, propionate; and Val, valerate); Ethanol (mg L^−1^), carbohydrates (C) (mgCOD L^−1^), proteins (mgCOD L^−1^) (P), and pH. (**b**) The relative abundance of sequencing reads at taxonomical genera level (≥5% in at least one sample) is projected onto the NMDS ordination synthesising the chemical dissimilarity. Colours indicate the two OLR conditions.

The occurrence of different metabolic pathways involved in the production of short and medium chain fatty acids is clearly evident in the NMDS ordination plot (Figure 6). The highest caproate production was observed at a high OLR whereas at a lower OLR, a mixture of VFAs was obtained, including propionate that is only slightly produced at a high OLR.

In situ hybridisation analysis revealed the large occurrence of *Firmicutes* and *Actinobacteria*, showing a progressive enrichment of the latter group mainly at a high OLR. The presence of various microorganisms belonging to these phyla was previously observed in CE studies due to their capacity of fermenting sugars and producing short-medium chain fatty acids [4,39,68].

The analysis of the successional changes of the chain-elongating microbiome during operating time showed a different bacterial speciation at different OLRs. As shown in the NMDS plot, the reactor operating at a low OLR was characterised by the occurrence of *Streptococcus* species mainly involved in lactate production and the genera *Succiniclasticum* and *Pyramidobacter* known for their capability to produce propionic acid [69,70] (Figure 6). At a high OLR, the lactate-producing *Actinomyces*, *Atopobium*, and *Olsenella* species were found together with *Pseudoramibacter* reported in the literature for its capability to produce caproic acid from lactate [4,71].

Remarkably, the enzymes involved in lactate and MCFAs production were hosted by most of the reconstructed bins (Table 3). The metagenomics evidences suggested a production of Acetyl-CoA, a key intermediate in the RBO pathway, primarily from lactate. Indeed, the presence of lactate racemase, lactate permease, and lactate dehydrogenase enzymes in nine of the 11 bins annotated supports the potential involvement of the microbial community in lactate production. In addition, bins 1, 2, 3, 4, 5, 6, 7, and 8 were predicted to harbor the pyruvate ferredoxin oxidoreductase, the enzyme involved in the conversion of pyruvate produced from lactate to Acetyl-CoA [72]. The occurrence of alcohol dehydrogenase in the genomes of bins 1 and 2 might suggest ethanol as an additional or alternative substrate however, the absence of an acetaldehyde dehydrogenase enzyme did not support the ethanol involvement in the CE process at a high OLR. These findings are consistent with lactate production mainly by *Actinomyces* and *Olsenella* genera, as previously observed [4,71]. The RBO pathway includes the production of three intermediates (acetoacetyl Co-A, 3-hydroxybutytyl-CoA, and crotonyl-CoA) for the formation of butyryl-CoA [25]. Four enzymes are responsible for this pathway (acetyl-CoA acetyltransferase, 3-hydroxyacyl-CoA dehydrogenase, enoyl-CoA hydratase, and butyryl-CoA dehydrogenase) [25]. Although they produce different intermediates, the same set of enzymes can mediate the conversion of butyryl-CoA to caproyl-CoA which can be further transformed to caproate trough the butyryl-CoA:acetate CoA-transferase enzyme [73]. The genome of bin 1 harboured all of the above mentioned enzymes as a further evidence of the possible active role of *Pseudoramibacter alactolyticus* in the lactate-based production of caproate observed at high OLR. Furthermore, some of the key enzymes for the RBO pathway were found in the genomes of bin 4, 5, 6, and 7 suggesting a possible interplay of genera *Olsenella*, *Succiniclasticum*, and *Mogibacterium* in the CE process. Butyrate kinase enzyme was found in the genomes of bins 5, 7, and 10, highlighting the potentialities of *Olsenella*, *Mogibacterium*, and *Prevotella* to produce butyrate. None of extracted genomes revealed the enzymes involved in hydrogen production. Metagenomic analysis also revealed other possible metabolic pathways. For example, key enzymes responsible for succinate production (e.g., succinyl-CoA:coenzyme A transferase, succinate—CoA ligase, succinate dehydrogenase) were recovered in some reconstructed genomes of *Mesosutterella* and *Succiniclasticum* genera, suggesting their potential role in the production of this chemical. Succinic acid is of great interest in cosmetic, food, and pharmaceutical industries and research efforts are currently focused towards the optimisation of its biological production [74].

The bacterial composition and genomes description suggest a possible lactate-based CE process in the reactors herein considered. In agreement, eukaryotes such as *Saccharomyces cerevisiae*, *Kluyveromyces marxianus*, and *Pichia stipites*, previously used in similar studies for improving CE by producing ethanol [75,76,77,78], were not detected in this study. The eukaryotes found with high-throughput sequencing were most likely associated with the feed due to their known use in cheese production and their pathogenic activity against fruits and vegetables.

## 5. Conclusions

In conclusion, this study reported the production of caproate from real FW in a single-stage fermentation process without the addition of external electron donors. The OLR influenced the caproate production in terms of rate and yield, with the highest values obtained at the highest OLR. Despite ethanol availability, the process was carried out by highly selected microbial communities characterised by the absence of *C. kluyveri*, commonly associated with ethanol-based CE process. The process and biological data fully support the establishment of a stable long-term lactate-based CE.

## Figures and Tables

**Table 1:** Characterisation of the liquid extracts fed into fermenter operating at organic loading rate (OLR) 5 and OLR 15.

	OLR 5	OLR 15
pH	5.1 ± 0.2	4.7 ± 0.4
TS (g L^−1^)	18 ± 2	46 ± 2
VS/TS (%)	84 ± 2	87 ± 3
CODtot (gCOD L^−1^)	21 ± 1	60 ± 1
CODsol (gCOD L^−1^)	20 ± 2	59 ± 7
Soluble proteins (gCOD L^−1^)	7 ± 1	24 ± 6
Soluble carbohydrates (gCOD L^−1^)	13 ± 2	34 ± 8

**Table 2:** Identifiers, taxonomic affiliation revealed by SILVA database and BLASTn search of the amplicon sequence variants (ASVs) discussed in the study.

Code	ASV Identifier	SILVA Taxonomy	BLASTn Alignment	% id
ASV4	c75c112ff6b6952240dcb5867a886c17	Actinomyces	Actinomyces succiniciruminis strain AM4	100
ASV27	df1a5bef682db901e0210b69c9cb3b14	Olsenella	Olsenella umbonata strain DSM 105334	100
ASV33	78ffaee8546019891250020e65c68589	Olsenella	Olsenella sp. Marseille-P8424	100
ASV54	75ef19e4491800b0708581b8ffeacc0f	Bacteroides	Uncultured Bacteroides sp. clone H10_Plate183	100
ASV57	8b7509a609eb84d3987832ae77119cb7	Bacteroides	Bacteroides uniformis strain ClaCZ126	100
ASV255	19b01f54a2c0ecc05c1f83c93d63191a	Streptococcus	Streptococcus equinus strain CNU 77-41/Streptococcus bovis strain MPR4	100
ASV262	bceb7314e96d011cae23d202d36fcee2	Streptococcus	Streptococcus sp. strain ING2-D5A	100
ASV292	104aba61760a24ee1a701a3c127f1d26	Pseudoramibacter	Pseudoramibacter alactolyticus strain KCOM 3148	100
ASV295	f2e68526ccce01878fe0c40c48bd9c19	Pseudoramibacter	Pseudoramibacter alactolyticus strain KCOM 3148	99.6
ASV301	ce665cba504c954f79109c671c641d5e	Mogibacterium	Mogibacterium sp. Marseille-P3194	98.5
ASV379	c94cbbf6b3bd3ae962dce819cb0c4d7e	Catenisphaera	Uncultured bacterium clone ATB-KS-1018	100
ASV417	0f7b568246fca80e1032f2ecfa176027	Succiniclasticum	Uncultured bacterium	100
ASV423	545cf0eb7e9979e3dbffa5dedde96dc5	Succiniclasticum	Uncultured bacterium	100
ASV424	5ebd9d2a80bf337c65091121d673545d	Succiniclasticum	Uncultured bacterium	100
ASV426	75fa310e7f4b199275eb0863cdc2df52	Succiniclasticum	Uncultured bacterium	99.8
ASV434	b8e7175e165c8658c7931f20824acb78	Succiniclasticum	Uncultured bacterium	100
ASV442	ef7434e97c63ff86f4c41a5d0ab7b8b9	Succiniclasticum	Uncultured bacterium	100
ASV545	3731ccd38ed8b26b9aaf6fb01ddc2019	Sutterella	Uncultured bacterium clone ncd859g03c1	100
ASV550	1faf3f3f12e8ff309fdc9258c0437060	Sutterella	Uncultured bacterium clone ncd859g03c1	99.6
ASV555	a154945f5bed95aa6f4f44f1c35a4538	Sutterella	Uncultured bacterium clone HLT4_256	100
ASV584	55247c4ddc469b05adb88ed7696bce70	Pyramidobacter	Pyramidobacter piscolens strain KCOM 1858	100

**Table 3:** Sequencing and assembly statistics and PROKKA annotation results. Contigs, number of contigs; Genome N50, the shortest contig length needed to cover 50% of the genome; genome size, the total length of each bin; GC, the content (%) of guanine-cytosine (GC) nucleotides; total coding sequences (CDS), number of predicted CDS; matching to COGs (Clusters of Orthologous Groups), number of CDS in COG classification; missing CDS, number of CDS not classified in COG.

BIN No.											
	1	2	3	4	5	6	7	8	9	10	11
GTDB taxonomy	*Pseudoramibacter Alactolyticus*	*Mesosutterella multiformis*	*Actinomyces succinicuruminis*	*f_UMGS124*	*Olsenella umbonata*	*Succiniclasticum sp900316935*	*Mogibacterium timonense*	*Lachnospira rogosae*	*Lactobacillus delbrueckii*	*g_Prevotella*	*Bifidobacterium thermophilum*
Contigs	170	14	174	40	174	59	80	20	103	105	15
Genome N50	24,510	228,379	37,014	115,064	22,034	74,578	155,420	203,601	29,461	51,304	218,912
Genome size	2,119,067	2,591,130	3,063,980	3,128,073	1,972,600	2,068,360	2,295,636	2,974,580	1,854,049	3,368,755	2,232,969
GC (%)	53	57	70	69	66	59	53	36	50	51	60
Completeness (%)	99.1	96.9	99.0	99.2	87.9	93.9	99.3	98.7	98.4	99.3	100.0
Contamination (%)	1.64	0.00	1.94	0.69	0.81	0.30	0.00	0.67	0.00	0.68	0.00
Total CDS	2028	2254	2447	2459	1736	1811	1888	2665	1764	2469	1699
Matching to COGs	822	962	1005	767	628	751	658	865	727	746	658
Hypothetical protein	812	877	1065	1199	774	688	816	1337	688	1211	770
With Enzyme CommissionNumber	250	212	242	301	228	225	269	294	209	275	173
Missing CDS	144	203	135	192	106	147	145	169	140	237	99
Total tRNA	49	49	46	61	51	49	46	48	70	49	49
Total rRNA	2	3	0	5	1	1	4	1	7	5	0

**Table 4:** Detailed information and presence of functional enzymes involved in acetyl-CoA formation, reverse β-oxidation, energy conservation, hydrogen formation, and butyrate formation in each bin according to Liu et al. [27]. +, present; −, absent.

Predicted Function	Enzyme Abbreviation	EC Number	Enzyme	BIN No.										
				1	2	3	4	5	6	7	8	9	10	11
Acetyl-CoA formation	LacR	5.1.2.1	Lactate racemase	+	+	−	−	+	+	−	−	−	−	−
	lacP	2.A.14	Lactate permease	−	−	+	−	−	−	−	−	−	−	−
	LDH	1.1.1.27	Lactate dehydrogenase	+	−	+	−	+	+	+	+	+	−	+
	PFOR	1.2.7.1	Pyruvate ferredoxin oxidoreductase	+	+	+	+	+	+	+	+	−	−	−
	ADH	1.1.1.1	Alcohol dehydrogenase	+	+	−	−	−	−	−	−	−	−	+
	ADA	1.2.1.10	Acetaldehyde dehydrogenase	−	−	−	−	−	−	−	−	−	−	−
Reverse β−oxidation	ACAT	2.3.1.9, 2.3.1.16	Acetyl-CoA acetyltransferase	+	−	−	−	−	+	−	−	+	−	−
	HAD	1.1.1.157, 1.1.1.35	3-Hydroxyacyl-CoA dehydrogenase	+	−	−	+	+	+	−	−	−	−	−
	ECH	4.2.1.150, 4.2.1.55	Enoyl-CoA hydratase	+	−	−	−	−	−	−	−	−	−	−
	BCD	1.3.8.1	Butyryl-CoA dehydrogenase	+	−	−	−	−	−	−	−	−	−	−
	EtfAB		Electron transfer flavoprotein A,B	−	+	+	−	−	−	−	−	−	−	−
	CoAT	2.8.3	Butyryl-CoA: acetate CoA-transferase	+	−	−	−	−	+	+	−	−	−	−
	ACT	3.1.2.20	Acyl-CoA thioesterase	−	−	−	−	−	−	−	−	−	−	−
Energy conservation	RnfABCDEG	7.1.1.1	Energy-converting NADH: ferredoxin oxidoreductase	−	−	+	−	−	−	−	−	−	−	+
	EchABCDEF		Energy-converting hydrogenase	−	−	−	−	−	−	−	−	−	−	−
H2 formation	H2ase	1.12.7.2	Hydrogen: ferredoxin oxidoreductase	−	−	−	−	−	−	−	−	−	−	−
Butyrate formation	PTB	2.3.1.19	Phosphate butyryltransferase	−	−	−	−	−	−	−	−	−	−	−
	BUK	2.7.2.7	Butyrate kinase	−	−	−	−	+	−	+	−	−	+	−
Others	BM	5.4.99.13	Butyryl-CoA: isobutyryl-CoA mutase	−	−	−	−	−	−	−	−	−	−	−
	ACOCT	2.8.3.19	Acetyl-CoA: oxalate CoA-transferase	+	−	−	−	−	+	−	−	−	−	−
	HadABC	4.2.1.157	(R)-2-hydroxyisocaproyl-CoA dehydratase	+	−	−	−	−	+	−	−	−	−	−
	CarC	1.3.1.108	Caffeyl-CoA reductase-Etf complex subunit CarC	+	−	−	+	−	+	+	−	−	+	−
	HypCDEF		Hydrogenase maturation factor	+	+	−	+	+	+	+	+	−	−	−

## Data Availability

The 16S and 18S rRNA gene sequences were deposited in the GenBank database (https://www.ncbi.nlm.nih.gov/genbank/) (accessed on 5 Frebruary 2021) under the accession numbers MW420990-MW421300 and MW433275-MW433567, respectively. This Whole Genome Shotgun project has been deposited at DDBJ/ENA/GenBank (https://www.ncbi.nlm.nih.gov/genbank/) (accessed on 5 Frebruary 2021) under the accession JADOBB000000000, JAEAMH000000000-JAEAMQ000000000 (bioproject PRJNA675427).

## Supplementary Materials

The following are available online at https://www.mdpi.com/2076-2607/9/2/327/s1, Figure S1: Protein and Carbohydrate removal (%) during the OLR 5 test. Figure S2: Protein and Carbohydrate removal (%) during the OLR 15 test. Figure S3: pH trend during the operation at OLR 5 (**a**) and OLR 15 (**b**). Figure S4: CARD-FISH images of the biomass reactor at different OLRs. (**a**) Same microscopic field after DAPI staining showing total cells in blue and members of phylum Firmicutes (LGC354abc probe) in green; (**b**) same microscopic field after DAPI staining showing total cells in blue and members of phylum Actinobacteria (HGC69a probe) in green. Scale bar = 10 μm. Table S1: Frequency heat-map of eukaryotic communities (≥1% relative abundance in at least one sample). The colour intensity in each cell shows the relative abundance.
