# Supplementary material for: Direct Conversion of Food Waste Extract into Caproate: Metagenomics Assessment of Chain Elongation Process
Source: Microorganisms. 2021 Feb 5;9(2):327. doi: 10.3390/microorganisms9020327 (PMC7915914; doi:10.3390/microorganisms9020327)
Supplement: Supplementary file 1 [file microorganisms-09-00327-s001.zip › microorganisms-1093881-SI/microorganisms-1093881-SI.pdf]

microorganisms

MDPI

Supplementary Materials: Direct Conversion of Food Waste Extract into Caproate: Metagenomics Assessment of Chain Elongation Process

Simona Crognale, Camilla M. Braguglia *, Agata Gallipoli, Andrea Gianico, Simona Rossetti and Daniele Montecchio

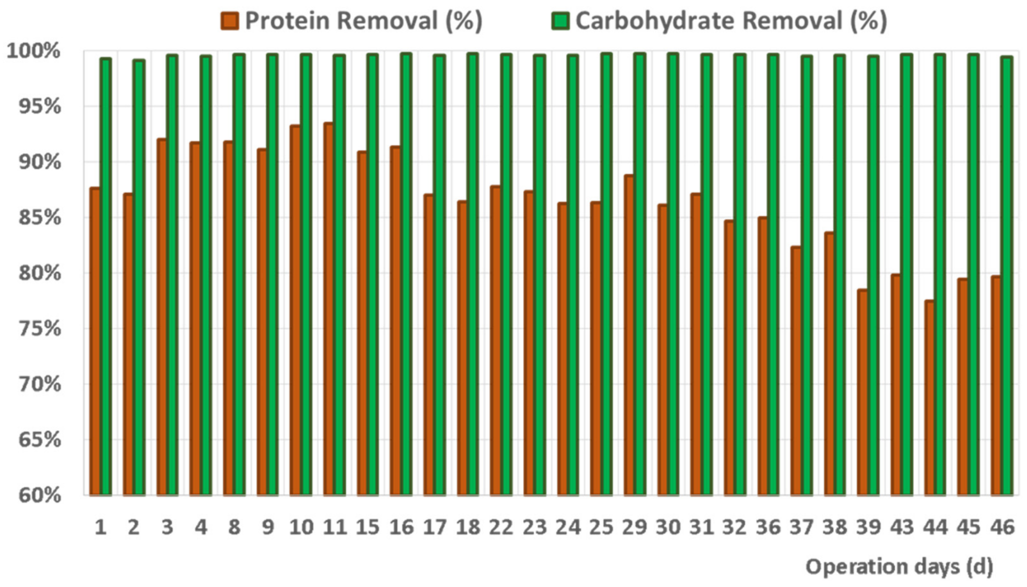

Figure S1. Protein and Carbohydrate removal (%) during the OLR = 5 test.

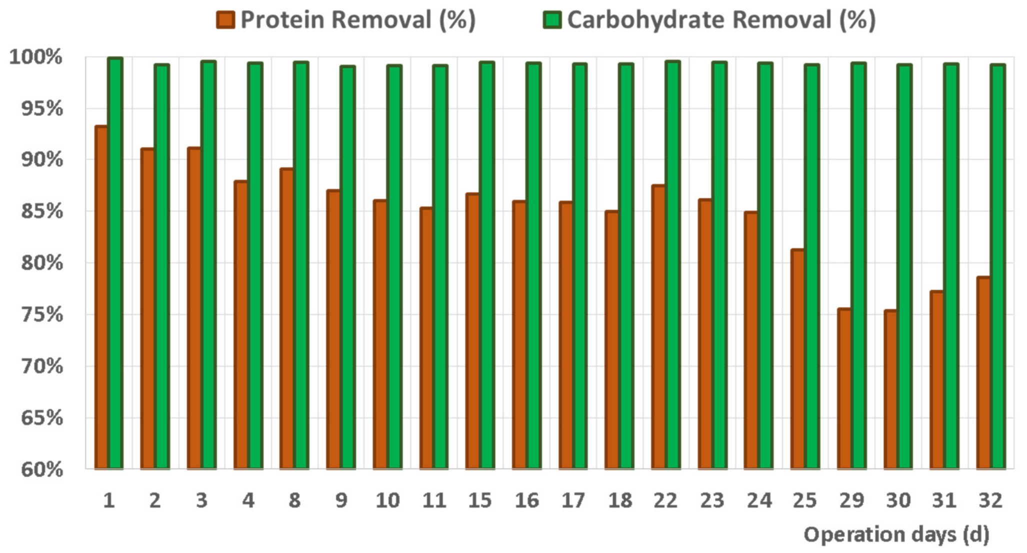

Figure S2. Protein and Carbohydrate removal (%) during the OLR = 15 test.

Publisher's Note: MDPI stays neutral with regard to jurisdictional claims in published maps and institutional affiliations.

Copyright: © 2020 by the authors. Submitted for possible open access publication under the terms and conditions of the Creative Commons Attribution (CC BY) license (http://creativecommons.org/licenses/by/4.0/).

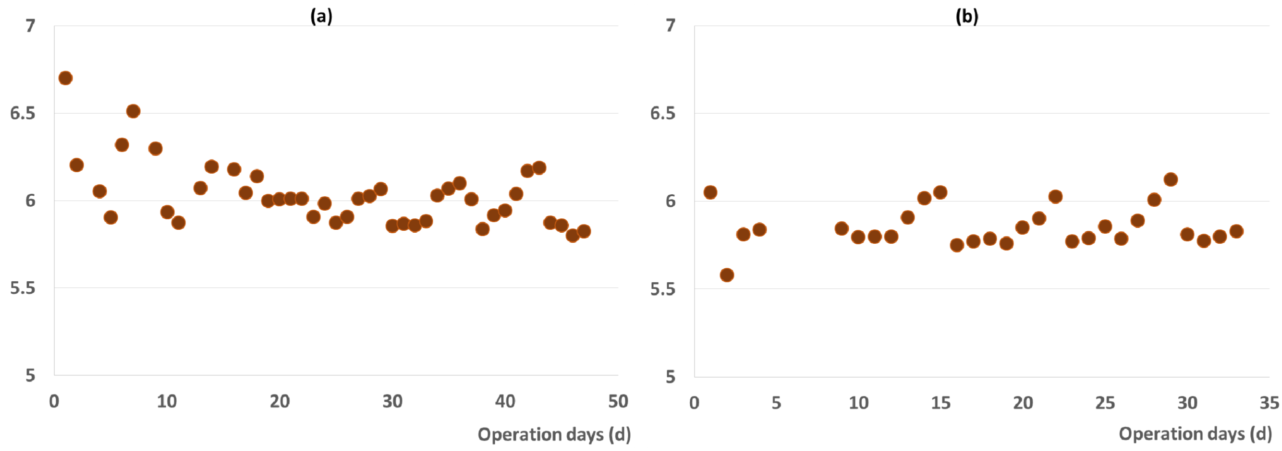

Figure S3. pH trend during the operation at OLR 5 (a) and OLR 15(b).

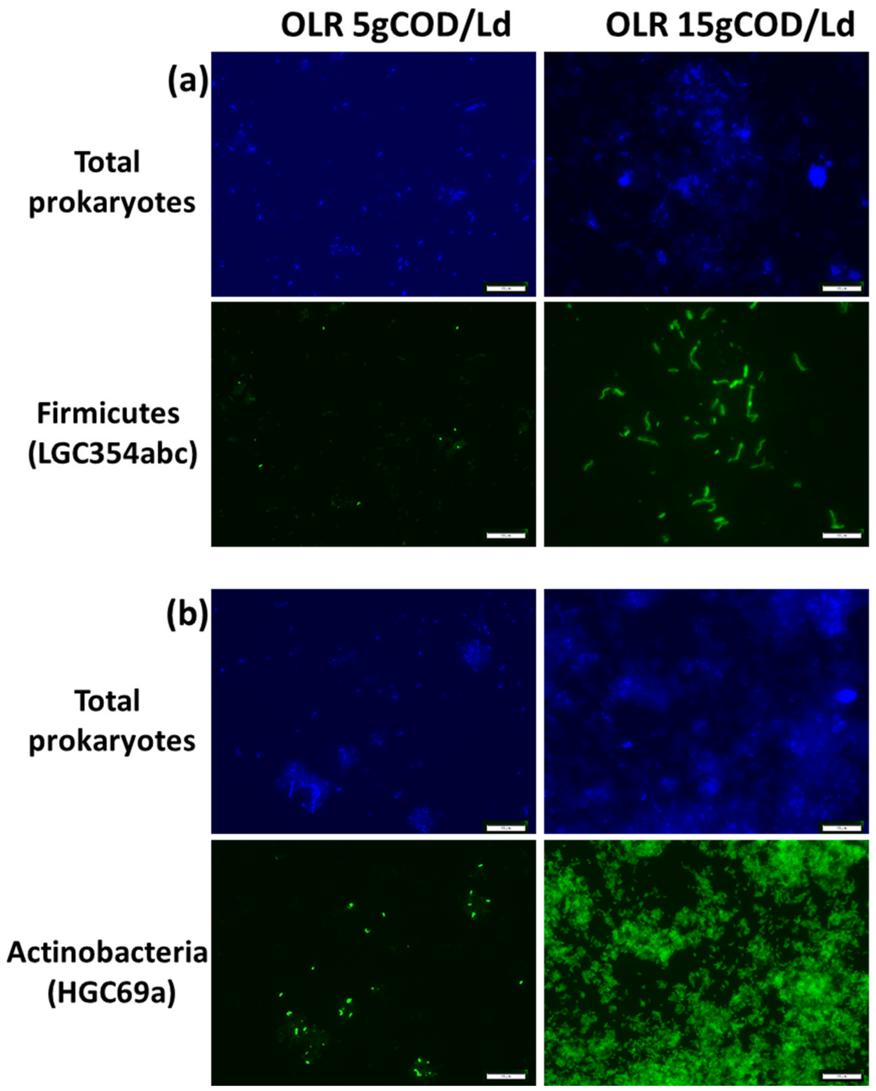

Figure S4. CARD-FISH images of the biomass reactor at different OLRs. (a) same microscopic field after DAPI staining showing total cells in blue and members of phylum Firmicutes (LGC354abc probe) in green; (b) same microscopic field after DAPI staining showing total cells in blue and members of phylum Actinobacteria (HGC69a probe) in green. Scale bar = 10 μm.
